# Development of New Lead-Free Composite Materials as Potential Radiation Shields

**DOI:** 10.3390/ma14174957

**Published:** 2021-08-30

**Authors:** Mansour Almurayshid, Yousif Alssalim, Farouk Aksouh, Rashed Almsalam, Meshari ALQahtani, M. I. Sayyed, Fahad Almasoud

**Affiliations:** 1 Nuclear Science Research Institute (NSRI), King Abdulaziz City for Science and Technology (KACST), Riyadh 11442, Saudi Arabia; malmurayshid@kacst.edu.sa (M.A.); yssalim@kacst.edu.sa (Y.A.); rmuslim@kacst.edu.sa (R.A.); mmalqahtani@kacst.edu.sa (M.A.); or fmasaud@gmail.com (F.A.); 2 Physics & Astronomy Department, College of Science, King Saud University, Riyadh 11451, Saudi Arabia; Faksouh@ksu.edu.sa; 3 Department of Physics, Faculty of Science, Isra University, Amman 11622, Jordan; 4 Department of Nuclear Medicine Research, Institute for Research and Medical Consultations (IRMC), Imam Abdulrahman Bin Faisal University (IAU), Dammam 31441, Saudi Arabia; 5 Department of Soil Sciences, College of Food and Agricultural Sciences, King Saud University, Riyadh 12372, Saudi Arabia

**Keywords:** shielding materials, radiation protection, silicon, polymer

## Abstract

Utilizing a polymer-based radiation shield offers lightweight, low cost, non-toxic compared to lead and solution for eliminating generated secondary neutrons. Incorporating silicon (i.e., one of the most abundant elements) in new applications, such as shielding, would have an impact on the economy and industry. In this study, seven potential shielding materials, composed of silicon, silicon carbide, and boron carbide embedded ethylene vinyl acetate (EVA) copolymers, are proposed. The shielding performance of these composite materials, including the attenuation coefficients (*µ*), the mass attenuation coefficients (*µ_m_*), the half value layer (HVL), the mean free path (MFP), and the radiation protection efficiency (RPE) were examined using photon beams. Measured *µ_m_* were verified against the calculated values. The averaged agreement was within ±7.4% between the experimental measurements and the theoretical calculation results. The HVL and MFP measured values for the polymer composites were lower than that for the pure EVA polymer, indicating the fillers in the polymers enhanced the shielding performance. The EVA + SiC (30%) and EVA + Si (15%) + B_4_C (15%) composites required the lowest thickness to stop 50% of the incident photons. The evaluation of experimental results of the RPE revealed that the polymer composites containing SiC (30%), Si (15%) + B_4_C (15%), or SiC (15%) + B_4_C (15%) succeeded in blocking 90–91% of X-rays at nearly 80 keV. However, a thicker shield of the proposed composite materials or combined layers with other high-Z materials could be used for higher energies.

## 1. Introduction

Controlling the vast usage of radiation in different daily applications is essential to avoid any harmful effects [1]. During practical procedures, an appropriate thickness of large atomic number (Z) material can offer sufficient shielding from radiation beams. Lead (Pb) is a widely used material for the construction of photon shields, especially for low-background applications [2]. The high cost of pure Pb and the issue of its toxicity can form constraints in many shielding applications. As an alternative to metals, concrete is often used in the construction of large-volume shields, due to its good availability and low cost [3,4]. However, the drawback of the direct irradiation of concrete is that activation of radioactive materials could occur post-use, due to its composition, and these materials could include ^40^K and radioactive fallout products [5,6]. Shielding from neutrons and secondary products of neutron interactions is crucial and requires a shielding material with high absorption cross sections. Therefore, low-Z elements or materials are the most active moderators, such as polymers considered as hydrogen-containing materials. One of most commonly available polymers is high-density polyethylene (HDPE), which is used in numerous applications. Another polymer that can be used as a moderator is ethylene vinyl acetate (EVA), which offers flexibly, a low manufacturing cost, and toughness [7]. In order to generate a radiation shielding material suitable for gamma radiation and neutrons, researchers have attempted to prepare polymer-based composites mixed with elements or materials and examine and characterize their radiation attenuation ability. For example, the possibility of using an X-ray shield based on a Pb-free HDPE composite material has been examined [8]. The filler materials were tungsten (W) and molybdenum (Mo), added in different percentage to the HDPE. The result was promising, as there was a clear impact on the attenuation of the beam from 50–250 keV. In addition, the 15% filler concentration in HDPE had a higher shielding efficiency than that for 5% and 10%. 

In this study, different Pb-free fillers embedded in EVA polymers were used. The fillers were selected for the following considerations. Silicon (Si) (Z = 28) is considered the second most abundant element on the planet in solid form and is produced commercially from sand [9,10]. In the desert landscape of Saudi Arabia, Nash et al. found 45.31% silicon in samples from the Ad Dahna’ Desert and 39.44% silicon in those taken from the Rub’ al Khali Desert. Utilizing this widely available Si in new applications would make a significant contribution to the development of industry and the economy [9]. Silicon is non-toxic in all its natural forms, it allows for flexible fabrication, and possesses a good interaction cross-section compared to Pb. For instance, the calculated mass attenuation coefficient at an energy of 661.7 keV is 0.07 cm^2^ g^−1^ for Si and 0.10 cm^2^ g^−1^ for Pb [11]. Therefore, it could be suitable for applications that require a lightweight radiation shield. In this study, silicon, in the form of pure Si and silicon carbide (SiC), was embedded in a 70% EVA polymer to improve the polymer’s shielding properties. In addition, study samples containing 15% and 30% of high capture cross section material, for neutrons such as boron, in the form of B_4_C were incorporated into an 70% EVA polymer and investigated for the attenuation of photon radiation [5,12,13]. The attenuation efficiency for the composite materials was evaluated by analyzing their effects on the attenuation coefficient (*µ*), mass attenuation coefficient (*µ_m_*), half value layer (HVL), and mean free path (MFP).

## 2. Materials and Methods

### 2.1. Definitions

The effectiveness of a potential shielding material towards a radiation beam can be examined according to many shielding parameters, with consideration of chemical composition, energy, and penetration thickness [14]. One measure that can be calculated is the linear attenuation coefficient (*µ*), which is an estimate of the remaining intensity (*I*) compared to the initial intensity (*I_o_*) of a beam after passing through a thickness of absorbent material (*x*) measured in cm, as shown in Equation (1) [15,16]:

(1) $$I=I_{o}e^{-\mu x}$$

To validate the experimentally measured results, comparisons can be made with the theoretically generated results of the mass attenuation coefficient *µ_m_* of shielding materials; this is usually used to provide the variation of μ with the density ρ of the absorber as follows:

(2) $${µ}_{m}=\frac{\mu }{\rho }\;\frac{{\mathrm{cm}}^{2}}{g}\;\;$$

When dealing with a compound or mixture of elements, *µ_m_* is expressed as the following sum [7]:

(3) $$\;{\left(\frac{\mu }{\rho }\right)}_{c}=\Sigma_{i}\omega_{i}{\left(\frac{\mu }{\rho }\right)}_{i}$$

where the $\omega_{i}$ factors represent the weight fraction of element i in the compound.

Another value that could be used to illustrate the attenuation ability of a shield is the half value layer of the absorber (HVL), defined as the thickness that reduces the intensity of a beam to one-half its initial intensity as follows [14]:

(4) $$\mathrm{HVL}=\frac{\ln2}{\mu }=\frac{0.693}{\mu }$$

The mean free path (MFP) is also a concept related to *µ* (Equation (5)), defined as the average distance a photon beam traverses an absorber before it makes an interaction:

(5) $$\mathrm{MFP}=\frac{1}{\mu }$$

Low HVL and MFP measurements for an absorber suggest good shielding and attenuation abilities.

A quantitative analysis of the composite samples was made, namely radiation protection efficiency (RPE), using intensity values measured with and without samples, using [16]:

(6) $$\mathrm{RPE}=\left(1-\frac{I}{I_{o}}\right)\times 100\;\;\;\;\;\;\;\;\;\;\;\left(\%\right)$$

### 2.2. Sample Preparation

**Figure 1:**
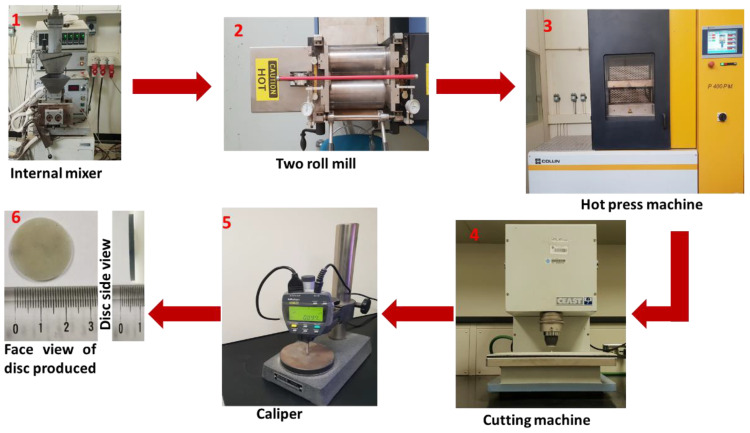
Demonstration of the composites based on a EVA polymer production procedure in the form of discs; the discs produced had a diameter of 25 mm and thickness of 2 mm.

The preparation process was conducted by mechanical means in an environment uncontaminated by other metals. The preparation process started by preheating the EVA copolymer (obtained from Sipchem company, Khobar, Saudi Arabia) in the chamber of a Brabender Plasticorder mixer (Brabender, Duisburg, Germany) to a temperature of 150 °C for approximately 3 to 4 min, allowing the polymer to melt. Subsequently, fillers were slowly added to increase the homogeneity of the composite. The screw mixer speed was increased to 60 rounds per minute (rpm) with a temperature of 150 °C for 10 min. The composite was then moved to the two roll mills, which were operated at 180 °C and 20 rpm for 5 min to ensure uniform mixing characteristics and to get a plain sheet. The composite sheet was then put in a steel frame with a thickness of 2 mm. This frame was placed in a hot press machine (Collin P 400 PM, Ebersberg, Germany) to obtain a flat sheet, from which discs were finally cut using a cutting machine (Ceast, Pianezza, Italy). The thickness of the disc was verified using a caliper (Mitutoyo, Aurora, IL, USA). Figure 1 illustrates the dimensions of one disc and the different process steps. 

Table 1 shows each polymer composite prepared, weighed using an analytical balance (Sartorius, Goettingen, Germany), with accuracy of 0.01%. The composite samples were given codes as follows: A, B, C, D, E, F and G, respectively. Sample A contained only EVA without any filler. All the prepared composite materials were based on 70% EVA. The remaining 30% was comprised of fillers; Si in sample B, SiC in sample B, and B_4_C in sample D. Other scenarios investigated the remaining 30% of the polymer composite being a mixture of two fillers such as Si 15% + B_4_C 15% in sample E, and SiC 15% + B_4_C 15% in sample G. 

### 2.3. Experimental Setup

**Figure 2:**
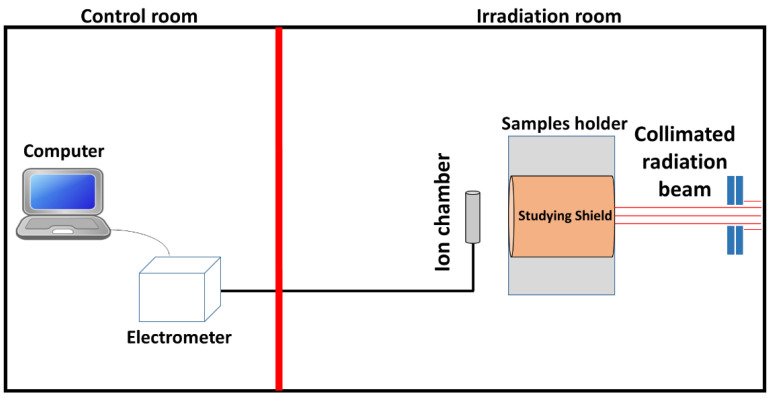
The radiation transmission experimental setup, showing the radiation beam passing through the studied samples and detected by the ionization chamber.

The experiment was performed in the radiation calibration lab at King Abdulaziz City for Science and Technology (KACST), Saudi Arabia. The experimental setup is sketched in Figure 2. The lab consists of a control room and an irradiation bunker separated by a thick Pb door. The main goals were to determine the effectiveness of seven different polymer composite materials on attenuating the photon beams. 

A ^137^Cs gamma source (661.7 keV) and an X-rays source (40.9–79.7 keV) were used to deliver radiation beams through a collimator to the sample placed in the holder 100 cm away from the radiation source. The whole procedure was controlled remotely from the control room. A sample holder made of Pb was put between the radiation source and the detector. The radiation beams transmitted through the discs were measured via a PTW 23,361 ionization chamber detector. An electrometer (PAM, Budapest, Hungary) in the control room, was linked to the ionization chamber detector to process the signal. The ionization chamber and electrometer were controlled using a computer using the Pico Amper Meter W2006 (PAMW2006) software package. 

### 2.4. Investigation of Shielding Efficiency for the Studied Composite Materials

The shielding efficiency of the composite materials prepared in the form of discs was tested by exposing 60 discs of each composite, with a thickness of 2 mm each, to radiation beams. The measurement procedure began by assuming *I_o_* is the measured incident radiation intensity without the discs. Sixty discs were positioned in the sample holder in the central beam axis to measure the transmitted intensity (*I*). Hence, the relative dose (%) can be determined by knowing the *I_o_* and *I*. From Equation (1), the *µ* can be calculated as well as the *µ_m_*, HVL, and MFP. The above procedure was performed for each beam energy, from 40.9 to 79.7 keV, using the X-ray source and 661.7 keV using the ^137^Cs gamma source. This allowed plots to be generated for *µ*, HVL, and MFP against energy E. In addition, the measured *µ_m_* was compared to the calculated value with an XCOM calculator using the National Institute of Standards and Technology (NIST) database [11]. The statistical uncertainties and the mean were determined by three repeated measurements.

## 3. Results

**Figure 3:**
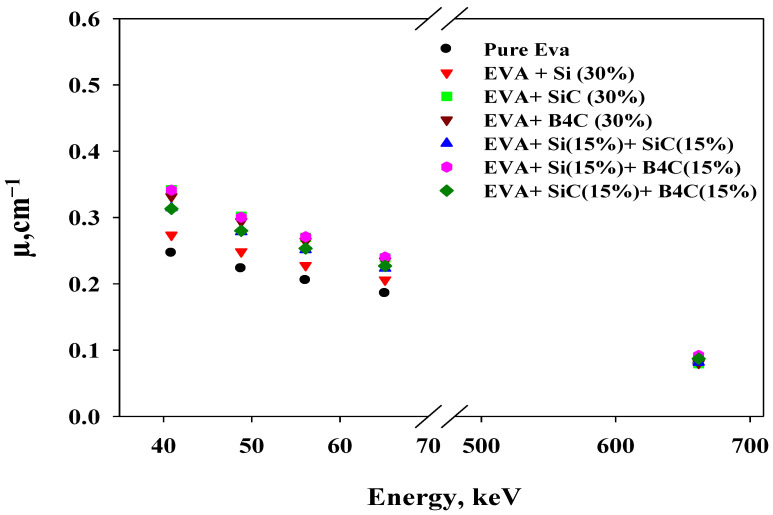
The experimentally measured *µ* values versus delivered energies in keV for the studied polymer composite samples. The error bars are smaller than the point size.

Solving Equation (1) as a function of *I*, *I_o_* and *x* allows the corresponding *µ* to be empirically determined for the proposed shielding polymer composites. Figure 3 shows the measured *µ* for the energy range used for the polymer composites prepared in this study.

The calculated *µ_m_* values of the proposed shields were determined from a NIST X calculator based on the NIST database [11]. Table 2 shows the measured and calculated *µ_m_* for different samples at an energy from 40.9 to 661.7 keV. 

**Figure 4:**
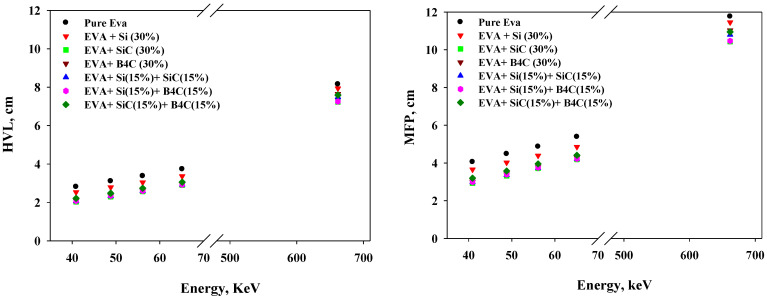
Variation of the measured HVL (**left**) and MFP (**right**) of the selected composite materials with the incident photon energies selected in this study.

Figure 4 shows important measurements of the attenuation abilities of the EVA-based composites across the energy range. In the figure, the HVL and MFP values display the overall rise with energy. In addition, the result shows that a higher thickness of pure EVA polymer was required to attenuate the radiation beam compared to the other composites.

The RPEs for the composite materials are shown in Table 3, indicating that the RPE value declined with the increase in incident energy. 

## 4. Discussion

The shielding efficiency of composite materials based on an EVA polymer was tested in attenuating 40.9–661.7 keV radiation beams. The composite materials were developed, mixed with either Si, SiC and B_4_C, or a mixture of the three, and coded A, B, C, D, E, F and G, as shown in Table 1, and eventually cut into discs. The measured attenuation coefficients *μ* were determined, and the plot in Figure 3 shows that it decreased with energy. This also shows that the samples with fillers had higher attenuation levels than the pure EVA polymer sample. The EVA + SiC and EVA + SiC + B_4_C shields possessed the highest reduction in transmitted radiation values below 100 keV. For all shields A to G, the attenuation reduction showed no intrinsic differences between the studied samples at 661.7 keV; becoming close, independent of the energy. The *μ* values varied highly according to the absorption and scattering mechanism of the X-rays; this variation depends strongly on the incoming photon energy and Z of the material [17]. Therefore, the attenuation efficiency for the shields with fillers was evident compared to the pure EVA in the lower energy region, where the predominant mechanism is photoelectric absorption [17,18].

The measured *µ* values for shields A to G reveal that the fillers in the polymer composites increased the attenuation efficiency, and this was evident below 100 keV in polymer composites containing SiC, B_4_C, Si + B_4_C, SiC + B_4_C, and Si + SiC. Among the polymer composites, the sample containing only Si had lower *µ* values. All the attenuation coefficients of the polymer composites were higher than shield A (EVA only). Although the impact of adding fillers to the EVA polymer could be greater, the reason might be that, for example, the percentage of the sample containing 70% EVA and 30% silicon could be expressed in terms of the number of moles ($n=\frac{m}{M}$), where m is the weight in grams, and M is the molecular mass ($M=\underset{i}{\sum }N_{i\;}M_{i}$, where $N_{i\;}$ is the number of element i atoms). Therefore, molecular mass is M = 114 g mol^−1^ for EVA (chemical formula (C_2_H_4_)_0.78_(C_4_H_6_O_2_)_0.22_) and M = 28 g mol^−1^ for Si, and hence $n=\frac{275.65\;g\;}{114\;g/\mathrm{mol}}=2.42\;\mathrm{mol}$ for EVA whereas $n=\frac{82.73\;g\;}{28\;g/\mathrm{mol}}=2.95\;\mathrm{mol}$ for Si, which is quite close to each other [19]. 

The measured *µ_m_* values are compared to the calculated values for the proposed polymer composites in Table 2, and the average agreement in *µ_m_* for shields A to G were 1.16%, 7.32%, 3.61%, 6.02%, 3.66%, 7.23%, and 12.19%, respectively. It was noticed that the agreement was excellent in the case of shield A (pure EVA), because there was no filler and the homogeneity of the material was high. In contrast, the uncertainty values increased due to the heterogeneity of the materials in the preparation procedure, affecting the result of the evaluation of *µ* values and eventually *µ_m_*.

To estimate the shielding ability of the composite materials, the HVL and MFP were calculated and plotted for the studied samples in Figure 4. It was observed that pure EVA had higher values of HVL and MFP, indicating the lowest attenuation ability among the materials. It can be seen that at 79.7 keV in the figure, the composite samples of SiC, B_4_C, Si + B_4_C, SiC + B_4_C, and Si + SiC reinforced EVA are below 0.8, the thickness of pure EVA required to absorb 50% of the photons. 

Different polymer composites have been examined in previous studies, mostly based on metals such as tungsten, and a comparison of the results obtained in this study to the literature was difficult because, to the best of the authors’ knowledge, no experimental research has synthesized and investigate composites based on EVA filled with Si, SiC, or B_4_C using low-energy X-ray beams. However, in the case of the Cs-137 energy, it was found that the *µ_m_* of the prepared EVA polymer was similar to the 0.084 cm^2^ g^−1^ obtained by Guo-hui et al. [20].

The experimental results of RPE show that a 12 cm-thick composite material could attenuate approximately 88–91% at 79.7 keV, making it a potential option for X-ray shielding in diagnostic radiology departments at hospitals using kilovoltage X-rays or research facilities using low energies [21].

Instead of increasing the thickness of shields made from composite materials, they could be enhanced by increasing the percentage of embedded materials. This would be an effective shielding option for facilities using energies below 661.7 keV. For illustrative purposes, if it is assumed that a composite material containing EVA (40%) mixed with Si (40%), SiC (10%) and B_4_C (10%) attenuated more X-rays and gamma radiation, the calculated *µ* at 661.7 keV would be 0.14 cm^−1^ for the proposed composite material compared to 1.26 cm^−1^ for Pb. Assuming Pb shields used in different thicknesses of 1.00 cm, 0.50 cm, and 0.25 cm were put separately in the path of the 661.7 keV, they would attenuate 72%, 47%, and 27%, respectively, using Equation (1). The required thicknesses for a similar attenuation of the proposed shield would be 8.60 cm, 4.30 cm, and 2.20 cm.

It is also possible to enhance the shielding efficiency of the proposed composites using a thin layer of a high-Z material (e.g., steel or tungsten) together with the studied shielding composite materials, to obtain sufficient shielding, especially for high-energy photon beams that may generate photo-neutrons, emitted from the interactions between photons and the nuclei of a high-Z material. In this way, the new materials could be used to reduce the dependence on the somewhat costly and toxic Pb shields, which requires reduction in some applications, for instance as a patient shield for low energy radiation beams and detector and electronic instrumentation isolation.

## 5. Conclusions

The aim of this study was to investigate the attenuation efficiency of different potential lightweight shielding materials based on a 70% EVA polymer content for X-rays (~40–80 keV) and gamma radiation (~662 keV). Values of *µ*, *µ_m_*, HVL, MFP, and RPE were obtained following a standard procedure using an ionization chamber detector to measure the intensities of transmitted radiation in the proposed shields. The outcomes of this study revealed that the fillers in the polymer clearly enhanced the attenuation ability, specifically as shown in the low photon energy range; having a low penetration power that can safely be stopped. The trend was generally that the *µ*, *µ_m_*, and RPE values decreased with photon energy, whereas the HVL and MFP values increased with photon energy, as expected. A future study direction could be utilizing the proposed materials to determine their performance for neutron shielding, especially the boron carbide composite. Furthermore, the homogeneity of the Pb-free composites (i.e., the dispersion quality of the fillers in the matrix) could be examined using different methods such as a scanning electron microscope (SEM), and this would enhance the differences between the measured and the calculated *μ_m_*. Instead of using a thick shield, the concentration of the fillers could be increased or a high-Z material layer could be incorporated into the shield to improve the gamma and X-ray shielding capability. In addition, materials based on silicon (being an abundant element) can be further examined, such as silicon with an amorphous structure, which is easier to produce, has a higher probability of interaction scattering centers, and is cheaper than crystalline silicon.

## Figures and Tables

**Table 1:** Composite sample list with component masses in g ± σ.

Sample	Density g cm^−3^	Polymer Composite Weight	EVA	Si	SiC	B_4_C	Total
A	0.94	g	276 ± 2.51	-	-	-	276
		%	100	-	-	-	100
B	1.15	g	193 ± 1.76	82.7 ± 0.75	-	-	276
		%	70	30	-	-	100
C	1.20	g	191 ± 1.74	-	81.9 ± 0.75	-	272
		%	70	-	30	-	100
D	1.16	g	192 ± 1.75	-	-	82.2 ± 0.75	274
		%	70	-	-	30	100
E	1.17	g	190 ± 1.73	40.8 ± 0.37	40.8 ± 0.37	-	272
		%	70	15	15	-	100
F	1.24	g	191 ± 1.74	41.0 ± 0.37	-	41.0 ± 0.37	273
		%	70	15	-	15	100

**Table 2:** The measured and calculated *µ_m_* expressed in cm^2^ g^−1^ versus incident photon beam energy for the EVA based polymer composites coded A, B, C, D, E, F and G.

**Energy (keV)**	**(A) Pure Eva**		**(B) EVA + Si (30%)**		**(C) EVA + SiC (30%)**		**(D) EVA + B_4_C (30%)**	
	**Measured**	**Calculated**	**Measured**	**Calculated**	**Measured**	**Calculated**	**Measured**	**Calculated**
40.9	0.2622 ± 0.0015	0.2308	0.2381 ± 0.0015	0.3238	0.2850 ± 0.0015	0.2874	0.286 ± 0.0015	0.2167
48.8	0.2373 ± 0.0011	0.2098	0.2163 ± 0.0015	0.257	0.2516 ± 0.0021	0.2364	0.2536 ± 0.0015	0.1986
56.1	0.2185 ± 0.0015	0.1978	0.1982 ± 0.0034	0.2239	0.2250 ± 0.0020	0.2109	0.2279 ± 0.0035	0.1880
65.1	0.1975 ± 0.0020	0.1876	0.1792 ± 0.0034	0.2002	0.1993 ± 0.0019	0.1923	0.2022 ± 0.0038	0.1789
73.6	0.1810 ± 0.0008	0.1804	0.1648 ± 0.0015	0.1863	0.1799 ± 0.0010	0.1810	0.1839 ± 0.0024	0.1724
79.7	0.1706 ± 0.0017	0.1762	0.1558 ± 0.0011	0.1790	0.1682 ± 0.0017	0.1750	0.1714 ± 0.0027	0.1684
661.7	0.0850 ± 0.0019	0.0860	0.0760 ± 0.0020	0.082	0.0670 ± 0.0025	0.0830	0.0770 ± 0.0033	0.0830
**Energy (keV)**	**(E) EVA + Si (15%) + SiC (15%)**		**(F) EVA + Si (15%) + B_4_C (15%)**				**(G) EVA + SiC (15%) + B_4_C (15%)**	
	**Measured**	**Calculated**	**Measured**		**Calculated**		**Measured**	**Calculated**
40.9	0.2860 ± 0.0034	0.3056	0.2689 ± 0.0021		0.2891		0.2467 ± 0.0011	0.2423
48.8	0.2536 ± 0.0022	0.2467	0.2379 ± 0.0033		0.2417		0.2204 ± 0.0018	0.2104
56.1	0.2279 ± 0.0015	0.2174	0.2147 ± 0.0025		0.2168		0.1995 ± 0.0010	0.1940
65.1	0.2022 ± 0.0025	0.1962	0.1909 ± 0.0018		0.1978		0.1788 ± 0.0024	0.1814
73.6	0.1839 ± 0.0027	0.1836	0.1734 ± 0.0010		0.1859		0.1631 ± 0.0027	0.1734
79.7	0.1714 ± 0.0035	0.1770	0.1635 ± 0.0017		0.1794		0.1532 ± 0.0013	0.1689
661.7	0.0690 ± 0.0028	0.082	0.0680 ± 0.0024		0.0830		0.0720 ± 0.0028	0.0820

**Table 3:** Measured radiation protection efficiency (RPE) values, with incident energy of the studied samples.

Energy (keV)	Pure EVA	EVA + Si (30%)	EVA + SiC (30%)	EVA + B_4_C (30%)	EVA + Si (15%) + SiC (15%)	EVA + Si (15%) + B_4_C (15%)	EVA + SiC (15%) + B_4_C (15%)
40.9	94.8	96.3	98.4	98.1	97.7	98.3	97.7
48.8	93.1	94.9	97.3	97.1	96.5	97.3	96.5
56.1	91.5	93.5	96.1	95.8	95.1	96.1	95.2
65.1	89.2	91.6	94.3	94.0	93.1	94.4	93.5
73.6	87.0	89.7	92.5	92.3	91.2	92.6	91.7
79.7	85.4	88.4	91.1	90.8	89.8	91.2	90.3
661.7	60.9	63.1	63.3	61.8	62.3	64.8	64.4

## Data Availability

Not applicable.
